# How Shall We Clothe Our Feet?

**Published:** 1874-12

**Authors:** 


### HOW SHALL WE CLOTHE OUR FEET?

We trust our readers will not feel that we intend to utterly wear them out by our constant allusions to this subject. We hope they take as much interest in the matter as we do. If they have followed us thus far in this discussion, we know they will not willingly allow us to hold our peace in future, until the race is redeemed from the awful thraldom imposed upon it since civilization's dawn. In fact, we know from our numerous correspondents and from the interest now widely manifested, that every household has its sufferers from this horrible foot-pestilence, and that no work upon which our JOURNAL has ever entered is as necessary, or will accomplish equal good. So we propose to continue sowing the good seed, assured that it will spring up and bear the rich fruit of amelioration for one of the most terrible evils suffered by the human race.

It appears that there are over one hundred million pairs of boots and shoes made in the United States each year. In all Europe there must be twice as many more. Saying nothing, then, about the number worn by the civilized people inhabiting other portions of the globe; making no account of those made use of by the semi-civilized and savage peoples of the earth—for, it is a fact that the foot-clothing worn by the savage is vastly more appropriate and less injurious than that worn by the refined and polite—we have the vast yearly aggregage of three hundred million pairs, or taken singly, six hundred millions of these hideous torturing machines called boots and shoes. Think of it for a moment! Reflect upon the enormous sum of torment and deformity induced by these terrible instruments of oppression! Remember that, until a very recent date, not one of all these millions of foot-coverings was constructed upon any correct principle, or could be worn without inflicting injury of a permanent nature, even though absolute pain was not induced in each and every case! Remember, too, that the world has, in effect, stood still for centuries, so far as rational development in foot-wear is concerned; while, in all other directions, it has progressed with tremendous and constantly accumulating force. Bear in mind that the wooden "last" upon which shoes are moulded or formed is the same to-day as it was centuries ago, with the exception of the varying width or length of that portion extending beyond the toes. Just here, at the toe, fashion comes in and decides the question of the amount of waste leather which each person shall carry about. Fashion does not reason; she is as blind and thoughtless as are the shoemakers who follow her behests. She utters her fiat, and the silly makers and venders obey her foolish whim with eagerness, even though her utterance is the death-knell of health and comfort to millions of human beings. She declares, perchance, that the females of the human race—the wives and mothers and sisters—must stand and walk perpetually on tip-toe, their heels elevated three inches in the air by the interposition of a slender cone, and the slaves of the shoe factory hasten to obey the atrocious mandate. Do they reflect that these high heels force the foot forward into the leathern sack and utterly destroy its symmetry, while imposing upon it the direst torture? Do they consider the list of fearful evils entailed upon woman by this elevation of a portion of her frame from nature's level; this transposition of the centre of gravity; this strain upon the muscles of the back; this enormous and continuous weight of the contents of the abdomen upon the uterine organs and the bladder? Do they know or care whether the race which they are doing so much to deform and destroy, pre-natally, is destined to be strong and vigorous, or weak and worthless, according to the method employed in clothing their feet? Clearly not. They know nothing, as a class, and care less. They know how to cut up leather and sew it and peg it into some arbitrary shape; but they do not stop to reflect that it were better, a thousand times better, for those who are to wear these wretched creations to wrap their feet loosely in rude skins, as the savages do, than to ruin health and banish comfort after the fashionable mode.

It was not our purpose to wage, in this chapter, the needed warfare against the high toppling heels. We shall come to that one of these days, and we will try to make the case so clear that the parent who thereafter allows the young girl to compel for herself a life of weakness and suffering and incapacity for the maternal function is guilty of criminal negligence. To-day our business is with a more serious evil, because it is a universal one, and because it is productive of disease and distortion to the entire family of civilized humanity. We allude to the form of the "last," by which is meant the block of wood upon which foot-clothing is moulded.

Upon this theme it is hard to speak with patience, so gross and so long-continued has been the ignorance concerning it. It is hard, too, to explain to the satisfaction of any person who is not an anatomist just wherein the world has erred in this direction, and how the error is to be remedied. But we have entered upon this work with a clear comprehension of the world's needs, and we propose to follow it up until the world's conversion from its present blindness is assured.

It will be admitted that the interior space surrounded by the leathern walls of a boot or shoe will be shaped the same as the "last" upon which that combination of thick and thin leather is moulded. There may be exceptions to this rule, as in the case of contraction of the leather after the "last" is withdrawn, or in the case of a shoe or boot which has been loosely "lasted"—that is to say, in which the leather has not been drawn closely to the "last;" in other words, the "stretch taken out" in the process of construction. This is exceptional; in most cases the "last" gives to the interior of the shoe or boot its own exact form. Of course, then, the great point is reached when we secure a properly-shaped "last." This "proper" form does not begin nor end with the width of the toe; the shape of the toe is perhaps the most insignificant incident in the formation of the structure. The great points to reach are, to see that the "last" is thick where the foot is thick; that the "tread" or sole of the "last" corresponds in locality to the "tread" or sole of the foot, and that the location of the instep of the foot is identical with the location of the instep of the "last." In each and all of these vital points the "last" in use for these many centuries, all over the civilized world, utterly fails.

Let us not be understood as saying that the "last" should simply be a reproduction of the human foot in wood. A shoe made upon such a last could not be worn, under all the varying circumstances of life. The shape of any natural foot depends, from moment to moment, upon the use to which it is applied. It is adapted to a multitude of conditions, and is slim or thick, or broad or curved, according to the duty imposed upon it. Its covering must be formed to meet these changes, as well as those of climate and temperature. To do this, and at the same time to secure comfort to the wearer, is not as simple a thing as many may suppose. It takes something more than the ordinary thoughtlessness or stupidity of the average shoe-maker and the average "last"-maker to fabricate such a covering.

A few years ago an intelligent mechanic, who had been studying upon the subject for over a quarter of a century, discovered that if the human foot were to be sawed lengthwise in two parts along the line of the instep, the saw would come out exactly on the inside of the great toe at one end and precisely in the centre of the heel at the other end. He found, also, that another line, drawn from the outside of the heel parallel with the first line, would strike about midway of the third toe and leave two-and-a-half toes and a very considerable part of the foot outside the said line. From these facts he reasoned that the universal system of placing the instep in the centre of the last was unnatural, and, further, that to so cut off the wood of the last as to practically deprive two or more of the toes and a large portion of the foot of any space, were enormities which ought not to be endured. So he made a pair of lasts in accordance with his discoveries, and the result has delighted that small portion of the world who have been informed concerning it.

This reform, the exact mechanical details of which we cannot now further discuss, is certain to work an entire revolution in the trade in boots and shoes. The world will not be tormented if it is once assured that torment can be avoided. That the system is correct; that all suffering is avoided by its use, and that the new boots and shoes, formed in accordance with nature's laws, as illustrated in the McComber "last," are beautiful and in every respect admirable, is our testimony, and, it seems to us, must be the testimony of all. By its general employment, a thousand evils, heretofore supposed to be inevitable, will be forever banished. Corn-cures and corn-doctors will no longer exist, for the need of them will cease. The horrible crippling of the feet will terminate, and man, and woman too, will thenceforth walk erect, without pedal distortion. It will take years to accomplish this, but it will surely be accomplished.

As we walked down Broadway a few days ago, we looked in at the elegant window of Lord & Taylor's palace—the window in which they display many specimens of ladies' boots and shoes, in showy colors, and elaborately stitched and ornamented. What vast good, we reflected, could not this great and honored house do, in behalf of poor, ignorant, suffering humanity, if it would only seek light on this most important topic, and declare that these distorting articles shall henceforth be kept only to look at and to warn, not to be worn! How many fashionable ladies would "rise up and call them blessed," if they would provide only goods made on the new last! How many, who are now little children, would some day seek them out and say, "you saved me from a life of misery by your properly formed shoes." How many devotees of fashion would express their joy at finding a shoe more beautiful than any other, while at the same time free from the power to give pain to the wearer.

Another point. If Lord & Taylor were to say that no goods not made on the McComber last should enter into their stock, in one year they would be doing more than nine-tenths of all the trade in ladies' and children's wear in this great city. It would not be possible to induce a lady to wear a distorting and painful and sprawling boot, after she had enjoyed the sweet experience of one correctly made. For ourselves, we could not be induced to accept the gift of the best pair of boots which could be made upon the old, erroneous last. In fact, it is difficult for us to endure the unequal pressure of some of our well-worn, old-fashioned boots, which were "as easy as an old shoe," before our feet were permitted to assume something like natural proportions by wearing our McComber boots.

For any prominent house which will enter upon the sale of goods made on the McComber plan, there is an enormous and speedy fortune in store. Better than this, there is a great service to be rendered to the human race. Whether shoe-dealers will see this, or care for it if they do see it, is doubtful. They are slow to see anything out of the old, time-worn grooves. They remember Plummer, of Portland, Maine, with his utterly mistaken plan of making projections on the sole of the last; or Miller, of

Boston, with his abominable idea of elevating the toe in the air at one end and the heel at the other, compelling the wearer to clump along on the balls of his feet; and they anticipate like failure for any innovation upon the old, brainless plan.

Somebody will see fame and fortune in the direction of retailing shoes made on the "McComber last," in New York City. It may be A. T. Stewart, or it may be Lord and Taylor or Arnold & Constable, or J. & C. Johnston, or some other of the great and successful mercantile establishments. To all these we would say, if you desire to look into the subject, we will gladly help you. We will do more; we will offer you convincing proof of the accuracy of our statements and the truthfulness of our conclusions. We will point you to those in the trade who use this system exclusively, and you shall see how rapidly they are gaining the business from their less thoughtful rivals. We will take you to W. Bristol, Jr. & Co., No. 8 Warren St., who make some thousand pairs of excellent shoes for ladies' and children each week on these lasts, nearly all of which they send to Massachusetts to a few retailers who know their superiority and cannot satisfy their customers or their consciences without them. This house is ready to supply the improved goods in any quantity, and to guarantee them for excellence of quality, elegance and comfort.

In conclusion we ask, will it be believed, that among the thousands of boot and shoe-dealers in New York City the house of Bristol & Co. alone manufacture and keep in stock these seasonable and elegant goods for ladies' wear? The idea surpasses belief, and forces us to remark that next month we mean to take up another phase of this all-important subject, and so continuing until we compel the attention of every respectable man and woman. Meantime, let us remark to the ladies among our readers, that if they cannot purchase the McComber boots and shoes in their own vicinity, they have but to write to Messrs. Bristol & Co., when their wants will be promptly supplied, at honest rates. The boots made on this plan will, as we can testify, greatly outwear those made on the old plan, because there is no great strain upon them and no breaking-in.

Let us warn our readers not to be deceived. They must not suppose they are buying the McComber goods, unless they find, plainly stamped on the sole of each shoe or boot, these words: "McComber Patent, Nov. 22, 1870." To stamp these words on goods not made on the improved plan is a felony, and the only assurance that the articles are genuine and worth having is the stamp alluded to.

